# Overcoming Health Inequities: Spatial Analysis of Seroprevalence and Vaccination Against COVID-19 in Chile

**DOI:** 10.1089/heq.2023.0204

**Published:** 2024-08-26

**Authors:** Muriel Ramírez-Santana, Juan Correa, Loreto Núñez Franz, Mauricio Apablaza, Paola Rubilar, Cecilia Vial, Lina Jimena Cortes, Juan Hormazábal, Luis Canales, Pablo Vial, Ximena Aguilera

**Affiliations:** ^1^ Departamento de Salud Pública, Facultad de Medicina, Universidad Católica del Norte, Coquimbo, Chile.; ^2^ Centro Producción del Espacio, Universidad de Las Américas, Santiago, Chile.; ^3^ Doctorado en Geografía, Pontificia Universidad Católica de Chile, Santiago, Chile.; ^4^ Departamento de Salud Pública, Facultad de Ciencias de la Salud, Universidad de Talca, Talca, Chile.; ^5^ Facultad de Gobierno, Universidad del Desarrollo, Santiago, Chile.; ^6^ Centro de Epidemiología y Políticas de Salud, Facultad de Medicina Clínica Alemana Universidad del Desarrollo, Santiago, Chile.; ^7^ Instituto de Ciencias e Innovación en Medicina, Facultad de Medicina Clínica Alemana Universidad del Desarrollo, Santiago, Chile.; ^8^ Facultad de Economía y Negocios, Universidad de Talca, Talca, Chile.

**Keywords:** spatial analysis, COVID-19, seroprevalence, vaccination, equity, Chile

## Abstract

**Background:**

In unequal economies, the spread of the first waves of the COVID-19 was usually associated with low socioeconomic status of individuals and their families. Chile exemplified this. By mid-2020, Chile had one of the highest SARS-CoV-2 infection rates in the world predominantly in poorer areas. A year later, the country launched a universal vaccination campaign based on the national strategy of immunization established in 1975. By 2022, Chile presented one of the highest COVID-19 vaccination coverages globally, reaching 94.3% of the population with the primary scheme by the end of 2022.

**Objective:**

This study analyzes the spatial distribution of SARS-CoV-2 seroprevalence at the beginning of the pandemic (2020) compared with the seroprevalence after 2 years of ongoing epidemic and COVID-19 vaccination campaigns (2022).

**Methods:**

Two population-based random samples of individuals aged 7 years and older from two Chilean cities were studied. Utilizing an enzyme-linked immunosorbent assay test, IgG antibodies were measured in serum of 1061 participants in 2020, and 853 in 2022.

**Results:**

Using the Global Moran's Index, the seroprevalence distribution pattern for the year 2020 showed clustering in the two cities. Conversely, seroprevalence and vaccinations were homogeneously distributed in 2022. These results show the success of the vaccination campaign in Chile, not only in coverage but also because it widely reached all individuals.

**Conclusions:**

The uptake of this preventive measure is high, regardless of the social and economic factors, achieving broad population immunity. The extensive deployment of the primary health care network contributed to reducing health inequities and promoting to universal health access.

**Figure:**
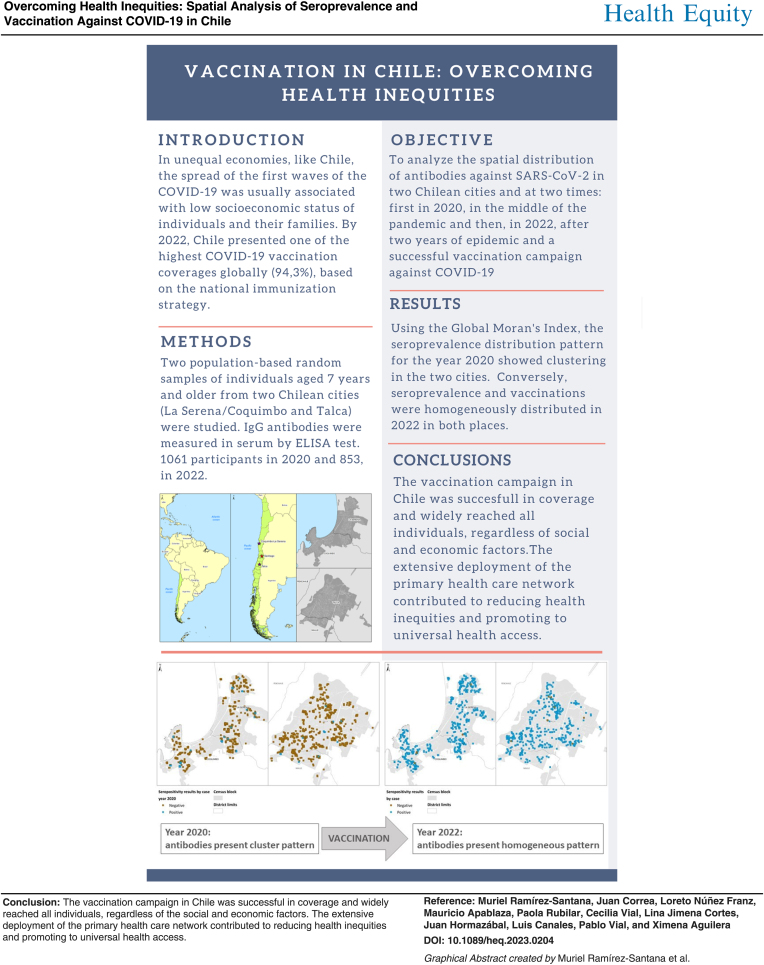

## Introduction

Social disparities and access to health have been a subject of study in epidemics before the emergence of COVID-19. This was evident during the 2009 H1N1 influenza epidemic.^1^ With the spread of the SARS-CoV-2 epidemic, it became clear that social factors influenced the risk of infection and inequality in access to health care.^2,3^ This situation was also evident in Chile.^4^ Race was one of the most studied factors in the United States,^5–7^ while the risk of death was also gender-differentiated.^8^ Subsequently, inequities in access to vaccination were also studied.^9^ Factors, including race,^10^ gender, education,^11,12^ geographic location,^13^ migrant status, and local language proficiency,^14^ influenced the vaccination uptake and final vaccination status of individuals across diverse territories.

Spatial analysis is a useful tool to study public health problems such as the territorial surveillance of diseases, outbreak dynamics, and distribution of public health policies.^15^ Furthermore, vaccination is a proven successful preventive strategy to tackle infectious diseases^16,17^ and seroprevalence studies are recommended for evaluating its implementation.^18^ Seroprevalence indicates the proportion of the population with antibodies to a given disease at a given time and can be generated by natural infection or by vaccination. In a scenario in which the SARS-CoV-2 pandemic has been very influential, researchers have reported the spatial relationship between the risk of SARS-CoV-2 infection and social health determinants in different areas of the world.^19^ In addition, spatial analysis of vaccination has been reported to evaluate the impact of several vaccines before the COVID-19 pandemic.^20–23^ Similarly, the spatial distribution of factors related to COVID-19 vaccination has also been recently studied in various countries.^24,25^

Chile had one of the highest vaccination coverages worldwide during the recent COVID-19 pandemic.^26,27^ Official data show that the overall coverage with the primary scheme of COVID-19 vaccination is 94.3% by the end of the year 2022, with 35% having additionally the fifth dose corresponding to the bivalent vaccine (against the original virus plus Omicron variant) by the end of 2023.^28^ After 2 years of a successful vaccination campaign, in 2022, a study reported that 98.4% of the population presented IgG antibodies in blood (seroprevalence).^29^ On the contrary, the seroprevalence due to natural infection was estimated to be 10.4% in the year 2020.^30^ That rate presented a heterogeneous territorial distribution, influenced by social variables such as education and population density.^30^ Areas with higher socio-educational vulnerability presented four to six times higher seroprevalence than wealthier neighborhoods.^30^ A similar link between the risk of infection and social determinants was established by previous literature.^4^

The proposed study hypothesis is that a high vaccination coverage with homogeneous distribution across the territory leads to the immunity being equally distributed. The objective of the study was to describe and analyze the spatial distribution of SARS-CoV-2 seroprevalence in the population of two Chilean cities, at the beginning of the pandemic and after 2 years of COVID-19 vaccination plan. Vaccination distribution was also analyzed in the year 2022.

## Materials and Methods

### Population context and sample of participants

**FIG. 1.**
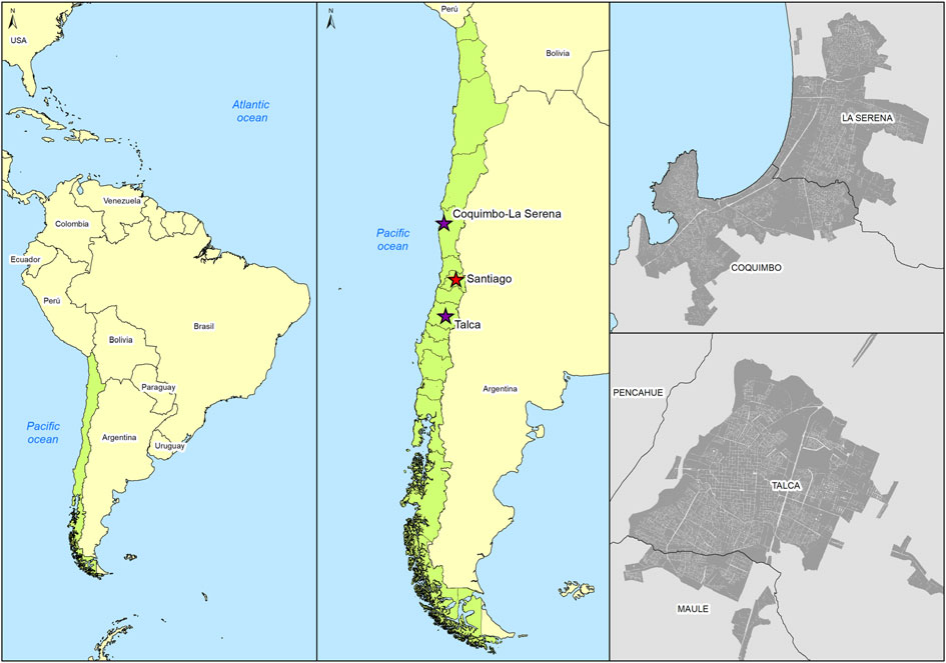
Study area: Chile and study locations (La Serena/Coquimbo conurbation and Talca City) in relation to Santiago (capital city of Chile).

Chile has a special geography, being a long and narrow country, in South America, on the coasts of the Pacific Ocean. Population-based seroprevalence studies were carried out to study the immune response against SARS-CoV-2 in three and two cities in Chile during the years 2020, 2021, and 2022.^29^ The research team was made up of researchers from three universities, one from Santiago City (the capital) and two regional universities, the first is located 450 km north of Santiago (Coquimbo) and the second is 250 km south to the capital (Talca). For a better understanding of the location, refer to the map in Figure 1.

Demographic and social data of the locations for the year 2020, according to the last Census of 2017, are shown in Table 1.^31^ Talca has a lower number of inhabitants and the poverty and overcrowding indicators are better than in the La Serena/Coquimbo conurbation.

**Table 1.** Demographic and Social Indicators of Chile and Studied Cities, Year 2020

Cities	Inhabitants (***n***)	Income poverty (%)	Multidimensional poverty (%)	Overcrowding households (%)
La Serena	249,656	10.2	20.1	15.6
Coquimbo	256,735	13.4	18.3	17.8
Talca	236,724	8.0	17.1	14.0
Chile	17,574,003	8.6	20.7	15.3

Source: Library of National Congress, Integrated Territorial Information System.

https://www.bcn.cl/siit/reportescomunales/comunal.html?unidad=0&anno=2020

A random population-based sample of 1061 people was selected in 2020 (Talca *n*=580 and La Serena/Coquimbo *n*=481). While in the year 2022, 395 individuals from Talca and 485 from La Serena/Coquimbo were studied (Total *n*=853). Both samples were selected using stages, stratification method, being census units the first stage, blocks the second, and households the last stage. Census units and blocks were listed and randomly selected. Finally, all residents older than 7 years were invited to participate in the study. The participants responded to a questionnaire after giving their consent and/or assent in the case of minors. Demographic, social, clinical, and vaccination variables were registered.

### Laboratory analysis

Participants also provided a blood sample, and IgG was measured in serum using the enzyme-linked immunosorbent assay (ELISA) test depending on availability. In 2020, an Elecsys immunoassay (Roche^®^ with a Cobas^®^ analyzer) was used.^30^ In the year 2022, the IgG antibodies were measured using an in-house validated ELISA.^32^ All test results are expressed as dichotomous variables (positive/negative). A lateral flow immunoassay (Livzon^®^, Cellex rapid test) was used for adults in whom venipuncture failed or was contraindicated and for young children who refused venipuncture. Further details about the sampling, field work and laboratory methods are reported elsewhere.^29,30,33,34^

### Spatial analysis

Individual data were georeferenced to the census block in which each address is located. For confidentiality purposes and protection of the participants' identity, the geographic location was randomized when designing maps. The spatial distribution of the seropositivity and the number of vaccines received was analyzed using ArcGis 10.7^®^ through spatial autocorrelation analysis (Global Moran's Index). Spatial autocorrelation is the term used to describe the presence of systematic spatial variation, showing the tendency of variable values' concentration or randomness dispersion through the space.^35,36^ The Global Moran's Index shows values between −1 and 1 for the statistically significant test. If the Moran's Index is positive, the spatial distribution of high values or low values in the space is more spatially concentrated than would be expected, under the null hypothesis (H_0_) of random spatial processes.

On the contrary, if the Moran's Index is negative, the spatial distribution of high or low values in the space is more spatially dispersed than expected. In the meantime, values near zero corroborate the null hypothesis (H_0_) of random spatial processes, that is, homogeneity.^37^

Based on the above, the assessment focused on analyzing seropositivity and vaccination coverage spatial patterns. In this way, H_1_ is that both seropositivity and vaccination have clustered spatial patterns, reflecting class biases and social determinants of health, showing important social spatial disparities. The hypothesis that studied variables show concentration (H_1_) is expected, as Chile presents social disparities that also affect the health inequities.^38,39^ Such premises were already demonstrated in relation to the COVID-19 risk of infection.^4,30,35,40^

### Ethical considerations

The research protocol was approved by the Ethics Committee of the Universidad de Talca and the Facultad de Medicina of the Universidad Católica del Norte, Statement numbers 50-2021 and 38-2021, respectively.

## Results

**FIG. 2.**
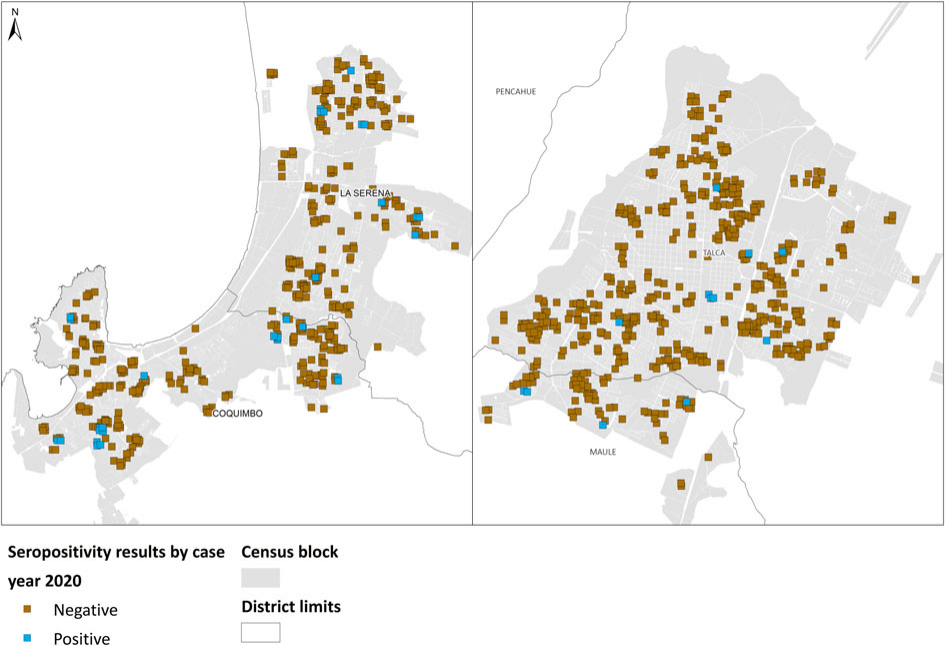
Distribution of seropositivity for COVID-19 in La Serena/Coquimbo conurbation and Talca City, year 2020. Shows the frequency of antibodies generated by natural infection. The Global Moran's Index analysis shows a clustered distribution.

**FIG. 3.**
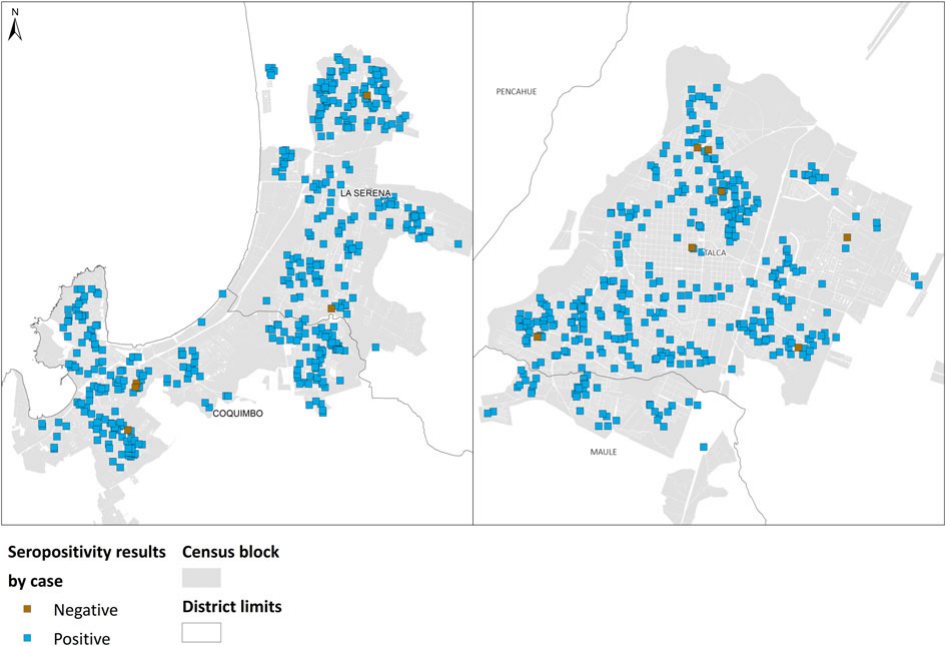
Distribution of seropositivity for COVID-19 in La Serena/Coquimbo conurbation and Talca City, year 2022. Shows the frequency of antibodies generated by vaccination and/or natural infection. The Global Moran's Index analysis shows a random distribution.

**FIG. 4.**
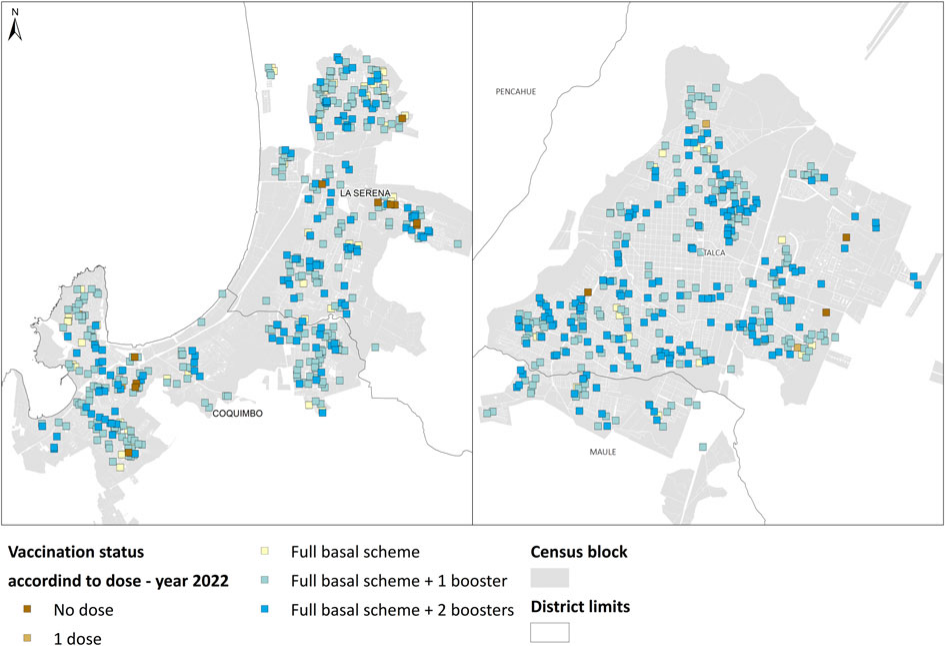
Distribution of vaccination against COVID-19 in La Serena/Coquimbo conurbation and Talca City, year 2022. The Global Moran's Index analysis shows a random distribution of the vaccine doses.

The spatial autocorrelation analysis shows clustered seroprevalence distribution in 2020, most prominently in the La Serena/Coquimbo conurbation. On the contrary, in 2022, both cities exhibited random distribution for seropositivity and vaccination. The results are presented in Table 2 and Figures 2–4. Then, the hypothesis of observing strong spatial inequalities in the distribution of seropositivity is accepted for year 2020, which reflects disparities in the risk of infection. However, this changed after 2 years, when seropositivity and vaccination coverage appeared randomly distributed in both cities. Therefore, the effect of socio-territorial disparities vanishes after 2 years of the vaccination plan.

**Table 2.** Spatial Autocorrelation of SARS-CoV-2 Seroprevalence and Vaccination Coverage in Two Chilean Cities (2020 and 2022)

City	Spatial autocorrelation (Global Moran's Index; 95% of significance)^a^		
	Seropositivity 2020	Seropositivity 2022	Vaccination coverage
La Serena—Coquimbo	Index: 0.137451 *p*-value: 0.01935	Index: 0.023 *p*-value: 0.21225	Index: 0.063707 *p*-value: 0.003178
Talca	Index: 0.03987 *p*-value: 0.000368	Index: 0.002813 *p*-value: 0.98947	Index: 0.040858 *p*-value: 0.048493

^a^ If Global Moran's Index tends to zero, then Ho is accepted, that is, the distribution is random or shows no patterns.

## Discussion

The spatial distribution of SARS-CoV-2 seroprevalence was analyzed together with vaccination variables in two different times. There are differences in the spatial distribution of SARS-CoV-2 antibodies between the first year of the epidemic (immunity by natural infection) and 2 years later (immunity generated by vaccination and/or disease. The seroprevalence distribution studied in the same Chilean cities in 2020, before the vaccination campaign, showed that the risk of infection was concentrated in vulnerable geographical areas, associated with lower education and higher population density.^30^ This effect is seen more clearly for the La Serena/Coquimbo conurbation, probably explained because it has worse poverty and overcrowding indicators than Talca, as shown in Table 1.^31^

Conversely, the 2022 seroprevalence was homogeneously distributed around the territories in both cities, showing that the vaccination campaign was successful not only in coverage, but also because it widely reached all individuals. At the same time, the consistent distribution of vaccine doses suggests a high acceptance of the COVID-19 vaccine in the Chilean population.

Since the beginning of the COVID-19 pandemic, no studies have reported the spatial distribution of seroprevalence after vaccination against SARS-CoV-2 infections. Nevertheless, several studies have reported the spatial distribution of public health events related to the COVID-19 vaccination. For example, these studies have explored at the distribution of vaccination centers,^24^ vaccines intake messages in social media, perceptions of the COVID-19 vaccine, mapping vaccine coverages^13^ and vaccination gaps, mutations of the virus, testing outcomes,^2^ modeling of the impact of preventing strategies, and the distribution of vaccines related to social factors.^41^ Among these factors, race has been one of the most extensively studied.^5,10,12,14^ The fact that the current seroprevalence is uniformly distributed across territories indicates that vaccination has led to widespread immunity, irrespective of economic and/or social factors.

Unlike in our study, the distribution of vaccination services presented significant geographical disparities in New Zealand,^42^ as well as the acceptance/hesitance of COVID-19 vaccines and the vaccine delivery in the United States.^12,25,43,44^ In Peru, demographic and social factors were related to the COVID-19 full vaccination coverage in children.^45^ Conditions such as living in urban areas, being poor and infant, having a mother with a low education level or was an adolescent at the time of birth, belonging to a large family, having no access to mass media, and few attendances to health control visits were inversely associated with full vaccination coverage.^45^

At the same time, the random distribution of vaccination found demonstrates that the vaccination campaign implemented in Chile was widespread, resulting in a strategy that reduces health inequities. Access to immunization and vaccination services depends on the health system that each territory or country has. Pressman et al. measured and promoted the development of a vaccine equity index,^9^ and Zhang et al. proposed to follow the strategy of spatial vaccination to achieve herd immunity.^46^ This sequential vaccination strategy covering different neighboring regions may reduce the risk of disease among regions, reducing at the same time the chance of developing vaccine resistance, and finally, the time required to cover large countries.^46^ Fortunately, the Chilean territory is not that extensive, the population is mainly urban, concentrated in large cities, and the public health system provides a good platform to perform successful vaccination campaigns.^47,48^

It is worth mentioning that Chilean immunization policies are universal, reaching both the beneficiaries of the public system and private insurance. In Chile, the vaccination process is carried out on the field by the vaccination teams from the local primary health care network (PHCN), and with the support of the health authority, represented by the Ministry of Health and the Regional Health Ministry Secretaries. The PHCN is administered by more than 300 counties and is composed of more than 2000 urban health centers and rural health posts along the country.^48^ This reality makes the immunization program to be universal.^47^ In fact, the national immunization program shows very high coverages even during the pandemic and the high uptake of vaccines derives from an ingrained health culture in our population.^49,50^

The main strength of this study is the use of randomized, representative, population-based samples, following the World Health Organization (WHO) recommendations for seroprevalence studies.^51^ Having studied two cities in Chile, excluding rural areas, may be a limitation when extrapolating the results to a whole country. However, with such a high national vaccination coverage and seroprevalences, it is to be expected that similar homogeneity will be observed in other cities in the country. Likewise, we are dealing with a northern city (La Serena/Coquimbo) and a southern city (Talca), both with populations of more than half a million people each.

## Conclusions

In emerging economies usually characterized by high levels of inequality, the spread of COVID is associated with the socioeconomic status of individuals. However, active target policies, such as universal vaccination, can reduce inequalities.

Changes in seroprevalence distribution from clustered to homogeneous, and random vaccination distribution, show that Chile executed a successful vaccination campaign against the SARS-CoV-2 infection. In addition to previous literature, we argue that the Chilean strategy achieved not only high coverage, but also ensured homogeneous reach across the entire population.

The use of spatial techniques could provide relevant information to evaluate health intervention strategies that are useful not only for pandemic outbreaks but also to monitor any health policy.

The extensive deployment of the local vaccination teams, organized under the PHCN, reduces health inequities, in terms of the risk of infection, and promotes universal health access.

## Abbreviations Used

ELISA: enzyme-linked immunosorbent assay
PHCN: primary health care network
WHO: World Health Organization
